# *SLCO3A1*, a Novel Crohn’s Disease-Associated Gene, Regulates NF-κB Activity and Associates with Intestinal Perforation

**DOI:** 10.1371/journal.pone.0100515

**Published:** 2014-06-19

**Authors:** Shu-Chen Wei, Yan-Yin Tan, Meng-Tzu Weng, Liang-Chuan Lai, Jen-Hao Hsiao, Eric Y. Chuang, Chia-Tung Shun, Deng-Cheng Wu, Ai-Wen Kao, Chiao-Shung Chuang, Yen-Hsuan Ni, Ming-Jium Shieh, Chien-Chih Tung, Yun Chen, Cheng-Yi Wang, Ramnik J. Xavier, Daniel K. Podolsky, Jau-Min Wong

**Affiliations:** 1 Department of Internal Medicine, National Taiwan University Hospital, Taipei, Taiwan; 2 Graduate Institute of Medical Engineering, National Taiwan University, Taipei, Taiwan; 3 Departments of Internal Medicine, Far Eastern Memorial Hospital, New Taipei, Taiwan; 4 Graduate Institute of Physiology, College of Medicine, National Taiwan University, Taipei, Taiwan; 5 Bioinformatics and Biostatistics Core, Center of Genomic Medicine, National Taiwan University, Taipei, Taiwan; 6 Graduate Institute of Biomedical Electronics and Bioinformatics, National Taiwan University, Taipei, Taiwan; 7 Department of Pathology and Forensic Medicine, National Taiwan University Hospital, Taipei, Taiwan; 8 Department of Internal Medicine, Kaohsiung Medical University, Kaohsiung, Taiwan; 9 Department of Internal Medicine, National Cheng Kung University, Tainan, Taiwan; 10 Department of Pediatrics, National Taiwan University Hospital, Taipei, Taiwan; 11 Deparment of Oncology, National Taiwan University Hospital, Taipei, Taiwan; 12 Department of Integrated Diagnostics and Therapeutics, National Taiwan University Hospital, Taipei, Taiwan; 13 Pediatric Surgery, Far Eastern Memorial Hospital, New Taipei, Taiwan; 14 Gastrointestinal Unit and Center for the Study of Inflammatory Bowel Disease, Massachusetts General Hospital, Boston, Massachusetts, United States of America; 15 UT Southwestern Medical Center, Dallas, Texas, United States of America; University of Saarland Medical School, Germany

## Abstract

**Background & Aims:**

To date, only one gene (*TNFSF15*) has been identified and validated as a Crohn’s disease (CD)-associated gene in non-Caucasian populations. This study was designed to identify novel CD-associated single nucleotide polymorphisms (SNPs)/genes and to validate candidate genes using a functional assay.

**Methods:**

SNPs from 16 CD patients and 16 age- and sex-matched control patients were analyzed using Illumina platform analysis. Subsequently, we expanded the study and followed 53 CD patients and 41 control patients by Sequenom MassArray analysis. Quantitative PCR and immunohistochemical staining were performed to assess mRNA and protein expression of the candidate gene on tissue isolated from CD patients. Genotype was correlated with CD phenotypes. Finally, the candidate gene was cloned and its effect on NF-κB activity assessed using a reporter luciferase assay.

**Results:**

*SLCO3A1* (rs207959) reached statistical significance in the first-stage analysis (*P* = 2.3E-02) and was further validated in the second-stage analysis (*P* = 1.0E-03). Genotype and phenotype analysis showed that the rs207959 (T) allele is a risk allele that alters SLCO3A1 mRNA expression and is associated with intestinal perforation in CD patients. Higher levels of mRNA and protein expression of SLCO3A1 were seen in CD patients compared with the control group. Overexpression of SLCO3A1 induced increased NF-κB activity and increased phosphorylation of P65, ERK, and JNK. Nicotine augmented the activation of NF-κB in the presence of SLCO3A1.

**Conclusions:**

*SLCO3A1*, a novel CD-associated gene, mediates inflammatory processes in intestinal epithelial cells through NF-κB transcription activation, resulting in a higher incidence of bowel perforation in CD patients.

## Introduction

Crohn’s disease (CD) and ulcerative colitis (UC), known collectively as inflammatory bowel diseases, are common chronic gastrointestinal diseases in the developed world. The diseases are particularly common in young people and have a major impact on their quality of life [1]. The pathogenesis of inflammatory bowel disease is complex, with both genetic and environmental factors contributing [1], [2]. The first susceptibility gene for CD was identified in 2001 [3], [4], initially named *NOD2* and later renamed *CARD15*. Although mutations in *CARD15* are strongly associated with CD in populations of European descent [3], [4], disease-associated *NOD2*/*CARD15* mutations are absent in Asian (Japanese, Korean, Chinese, Singaporean, and Taiwanese) CD populations and healthy controls [5]–[9].

Since the advent of human haplotypes by the International HapMap projects and the commercial availability of platforms that allow the testing of thousands of single nucleotide polymorphisms (SNPs) in a single genotyping reaction, the genome-wide association study (GWAS) has become a powerful and unbiased tool for detecting genetic risk factors by probing the whole genome and incorporating the statistical power of an association study [10]. Using this approach, the TH17 pathway gene *IL23R*, as well as the autophagy genes *ATG16L1* and *IRGM*, have been identified as CD susceptibility genes in patients residing in Western countries [11], [12]. Based on studies performed in populations from North America and Europe, meta-analyses and deep sequencing have led to the discovery of additional susceptibility genes/loci contributing to the risk of CD and/or UC [13], [14]. However, to date, only one gene, *TNFSF15*, initially reported from Japan [15], has been identified by GWAS in a non-Caucasian population. This gene was later confirmed to be associated with CD in other Asian countries [8], [16].

In parallel with the reported CD-associated genes identified in Western countries, we hypothesized that additional CD-associated genes exist in Asian populations. This study was therefore designed to identify novel Asian CD-associated genes using Illumina platform-based analysis. Since GWAS traditionally requires a large sample population to attain acceptable statistical power, one obstacle in performing GWAS in Asian countries is the comparatively low prevalence of CD. Though gradually increasing in recent years, the prevalence of CD was estimated to be 2 per 100,000 persons in Taiwan in 2008, approximately 11/100,000 in Korea, and approximately 21/100,000 in Japan, all much lower than the incidence in Western countries (approximately 200/100,000) [17].

To use a limited sample size without losing statistical power, we used independent samples in a two-stage experimental design, simultaneously decreasing the SNP number and increasing sample size at each stage. In the first stage, one group of patients was examined by genomic SNP genotyping microarrays (Illumina SNP genotyping Infinium II assay) to screen potential SNP candidates. In the second stage, an independent group of patients was examined by mass spectroscopy (Sequenom MassArray technology) to validate potential SNPs. Using this two-stage approach we identified *SLCO3A1* as a novel CD-associated gene and validated this finding through functional studies.

Over the years, several studies have shown that smoking is a risk factor for CD, but likely a protective factor for UC [18], [19]. A recent meta-analysis of GWAS showed that *SLCO3A1* is associated with nicotine dependence [20]. In our study, we provide evidence that nicotine induction leads to increased NF-κB activation in the presence of SLCO3A1, which might partially explain why smoking is an aggravating factor for CD.

## Materials and Methods

This study and the informed consent were approved by the Institutional Review Board of the Ethics Committee of the National Taiwan University Hospital (200906043R, 201212132RINB). Informed consent was obtained in all cases. For those under 18 years of age, the informed consent was obtained from the guardians on behalf of the children. The consent procedure was approved by the Ethics Committee of the National Taiwan University Hospital. The records of participants’ consent were locked and kept by the principal investigators following the guidelines set up by the Ethics Committee of the National Taiwan University Hospital.

After obtaining written informed consent, DNA was extracted from whole venous blood. For CD patients receiving endoscopy or surgery, tissue sampling was performed on the endoscopically identified ulcers (inflamed [I]) and endoscopically identified normal (non-inflamed [N]) tissue. Active disease was defined as active ulcers under endoscopy, while remission was defined as scar formation endoscopically. Mucosal samples were also collected from colorectal cancer as well as the colon from macroscopically and microscopically unaffected colonic areas of patients undergoing colectomy for colon cancer for normal colon control (control). A group of small intestinal tissue samples were collected from patients undergoing small bowel transplantation. Healthy donor intestinal tissue (normal) and grafts after at least three hours of reperfusion (reperfusion), made up the comparative samples for CD in the small intestine. All tissues were freshly frozen or immersed in optimal cutting temperature (OCT) compound (Ames Company, Elkhart, IN) and kept at−80°C until use.

### DNA Extraction and Hybridization for GWAS Array

Genomic DNA from 16 CD patients and 16 age- and sex-matched controls was extracted from blood by adding proteinase K-phenol-chloroform followed by 0.5% SDS and 200 µg/ml proteinase K. Illumina Human Omni1-Quad_v1-0_B SNP GeneChips (Illumina, San Diego, CA) containing 1,016,423 SNPs were used for the genome-wide assay according to the manufacturer’s instructions. To identify candidate SNPs for second-stage validation, quality control criteria were adopted. SNPs were excluded if (1) genotyping call rates were less than 90%, (2) minor allele frequencies (MAF) were less than 0.05, and (3) *P* values from the Hardy-Weinberg Equilibrium (HWE) test were greater than 0.05.

### Validation and Characterization of SNPs

In the second stage, 94 individuals (53 cases and 41 controls) were selected from the same Taiwanese cohort from multiple medical centers. The CD-associated SNP rs4263839 (*TNFSF15*) was used as a positive control in the second-stage validation. SNP genotypes were determined using the MassARRAY system from Sequenom (San Diego, CA) using the iPLEX protocol. PCR primers and extension primers were designed using SeqTool Document v1.0 (IBMS, Taiwan). The classification of SNPs was manually determined by MassARRAY Typer-Analyzer v3.3 software (Sequenom, San Diego, CA).

### Cell Culture

HEK293T cells and the human colon cancer cell line HCT116 were obtained from the American Type Culture Collection (ATCC, Manassas, VA). Cells were cultured in DMEM with 10% fetal bovine serum and 1% penicillin/streptomycin. Cells were grown at 37°C in a 5% CO_2_ atmosphere within a humidified incubator.

### Reagents and Antibodies

Polyclonal rabbit antibodies against phospho-p65, phospho-JNK, phospho-ERK 1/2, phospho-p38, phospho-AKT, total p65, JNK, ERK1/2, p38, and AKT were purchased from Cell Signaling Technology (Danvers, MA). Anti-SLCO3A1 was purchased from Abcam (Cambridge, UK). Other antibodies used were mouse monoclonal antibodies to FLAG and actin (Sigma, St Louis, MO) and nicotine was purchased from Sigma (St Louis, MO).

### Plasmids, Small Interfering RNA, and Transfection

FLAG-tagged human SLCO3A1 expression vector pcDNA4TAG-SLCO3A1 (FLAG-SLCO3A1) was generated by PCR amplification of SLCO3A1 cDNA, digestion with BamHI and XhoI, and insertion into the multiple cloning site of the pcDNA4-TAG vector (Invitrogen). HEK293T cells were transfected using Lipofectamine 2000 (Invitrogen) according to the manufacturer’s protocol.

### NF-κB Reporter Luciferase Assay

For NF-κB activity determination, cells were transfected with 20 ng pIV luciferase reporter plasmid and 0.05 ng *Renilla* plasmid. Activity was measured using the Dual-Luciferase Reporter Assay System (Promega, Madison, WI) in a BD Monolight 3010 luminometer (BD Biosciences, San Diego, CA) in accordance with the manufacturer’s instructions and normalized to *Renilla* activity.

### RNA Extraction and Real-Time RT-PCR

Total RNA from cell lines and tissue was isolated using an RNA extraction kit (Qiagen, Valencia, CA) according to the manufacturer’s instructions. For reverse transcription, 2 µg total RNA were transcribed using the iScript cDNA Synthesis Kit (Bio-Rad, Hercules, CA). Real-time (RT)-PCR was performed by a DNA Engine Opticon 2 (Bio-Rad) using iQ SYBR Green Supermix (Bio-Rad).

### Western Blot Analysis

Cells were lysed in NP-40 lysis buffer (50 mM Tris pH 7.5, 150 mM NaCl, 2 mM EDTA, 1% NP-40, 50 mM NaF, 1 mM Na_3_VO_4_, 10 mM Na_2_P_2_O_4_, Roche Complete Mini protease inhibitor) and centrifuged at 15,000 rpm for 20 minutes at 4°C. The supernatant was assayed for protein concentration (Bradford). Equal amounts of protein were solubilized in Tris-glycine SDS sample buffer (Invitrogen) and separated on 4–12% gradient Tris-glycine gels (Invitrogen). Following electrophoresis, proteins were transferred to polyvinylidene difluoride membranes and blocked with 5% bovine serum albumin in TBST (10 mM Tris, 150 mM NaCl, 0.1% Tween 20, pH 7.5). For MAP kinase antibody staining, membranes were blocked with 4% milk in TBST. Membranes were incubated with the specific primary antibody overnight, washed, incubated with appropriate secondary antibody conjugated to horseradish peroxidase, and developed using ECL (PerkinElmer Life Sciences). Membranes were stripped and re-probed with anti-total MAPK or anti-actin to confirm equal protein loading.

### Immunohistochemistry

Frozen sections (8 µm thick) were stained with the NoVo Link Polymer Detection System (Leica, Biosystems Newcastle Ltd, UK), followed by the AEC substrate kit (Vector Laboratories, Burlingame, CA), according to the manufacturer’s protocol. Tissues were counterstained with Mayer’s haematoxylin. An isotype antibody was used as negative control for staining. A pathologist, who was blinded to the genetic results, performed the reading of the immunohistochemical staining results.

### Statistical Analysis

Statistical analysis was performed with the R 2.15.1 package. Fisher’s exact test and logistic regression were used to investigate the association between individual SNPs and CD. Fisher’s exact test was used to determine the statistical significance of differences between case and control groups. Associations with risk of CD were estimated by odds ratios (ORs) and their 95% confidence intervals (CIs) using logistic regression with four different models including additive, recessive, dominant, and co-dominant models. Statistical differences between experimental groups were analyzed by Student’s *t* test. Data are expressed as means ± standard errors (SE). All experiments were repeated at least three times. *P* values less than 0.05 were considered to indicate statistically significant differences.

## Results

### Study Population

The first-stage Illumina platform analysis was composed of 16 CD patients and 16 age- and sex-matched controls. The second-stage Sequenom analysis was performed on 94 subjects, made up of 53 CD patients and 41 controls (Table 1). Clinical characteristics of CD patients are summarized in Table 2.

**Table 1:** Demographic data of populations in Illumina (stage 1) and Sequenom (stage 2).

Characteristic	1^st^ stage* (n = 32)		2^nd^ stage** (n = 94)	
	CD Patient(n = 16)	Normal control(n = 16)	CD Patient(n = 53)	Normal control(n = 41)
Sex				
Male	10	10	32	24
Female	6	6	21	17
Age				
Mean (Range)	29.1 (21–42)	29.1 (21–42)	34.5 (10–75)	39.6 (17–76)

*Illumina HumanOmni1-Quad_v1-0_B containing 1,016,423 SNPs.

**Sequenom MassARRAY system examining 38 SNPs.

**Table 2:** Clinical characteristics of CD Patients.

Clinical features	Total N = 53 (%)
Sex	
Female	21 (40%)
Male	32 (60%)
Age at Diagnosis	
A1: <16 yr	12 (23%)
A2: 17–40 yr	31 (58%)
A3: >40 yr	10 (19%)
Disease Location	
L1: Ileum:	17 (32%)
L2: Colon	11 (21%)
L3: Ileocolonic	25 (47%)
Disease Behavior	
B1: Inflammation	27 (50%)
B2: Stenosis	13 (25%)
B3: Perforation	13 (25%)
Surgery	
No	32 (60%)
Yes	21 (40%)

### rs207959 (T) Allele is Significantly Associated with Susceptibility to CD

The SNP rs207959, located in the intron of *SLCO3A1*, was a significant finding in the first-stage analysis (*P* = 2.3E-02) and was subsequently validated in the second-stage analysis (*P* = 1.0E-03). The positive internal control *TNFSF15* (rs4263839) had a *P* value of 1.5E-02 in the first-stage analysis and 3.1E-02 in the second-stage analysis by Fisher’s exact test. We further compared the allelic frequency and calculated the ORs of three different genotypes (TT, TC, and CC). The rs207959 (T) allele demonstrated significant susceptibility to CD (T vs C, OR = 3.46, *P* = 4.0E-4; TT+TC vs CC, OR = 3.8, *P* = 3.4E-3) (Table 3).

**Table 3:** Summary of association of SLCO3A1 between CD cases and controls.

SNP	Position*	Allele†		Genotype ofcase (%)				Genotype ofcontrol (%)				Trendtest	Allele 1vs 2		Genotype 11vs 12+22		Genotype11+12 vs 22	
		1	2	11	12	22	Sum	11	12	22	Sum		OR(95% CI)	P-value	OR(95% CI)	P-value	OR(95% CI)	P-value
rs207959	90456266	T	C	8	26	19	53	0	13	28	41	6×10^−4^	3.46	4×10^−4^	Inf	8.8×10^−3^	3.80	3.4×10^−3^
				(15.1)	(49.1)	(35.8)		(0.0)	(31.7)	(68.3)			(1.64–7.70)		(1.44-Inf)		(1.50–10.06)	

*Chromosome Position.

† Allele 1 is a risk allele.

CI: Confidence Interval.

### rs207959 (TT) Genotype is Associated with Intestinal Perforation in CD

Genotype and phenotype analysis of the rs207959 allele showed significant differences between gastrointestinal tract perforating (B3) and inflammatory phenotypes (B1) in patients with the T allele, which achieved a trend test *P* value of 0.0078 (TT OR = 1; CT OR = 0.03; CC OR = 0.04) (Table 4). However, there were no significant differences in other features, such as age of onset or disease location. These results suggested that the rs207959 (T) allele is a risk allele for causing intestinal perforation.

**Table 4:** Genotype and phenotype analysis of SLCO3A1 (rs207959).

SLCO3A1(rs207959)		11 (TT)		12 (TC)		22 (CC)		
(N = 53)		OR(95% CI)	P-value	OR(95% CI)	P-value	OR(95% CI)	P-value	Trend testP-value
Sex	Male vs Female	1	-	2.67 (0.52, 13.68)	0.4293	3.61 (0.64, 20.32)	0.2872	0.1672
								
Age at Diagnosis	A2 vs A1	1	-	0.67 (0.11, 4.22)	0.9876	1.22 (0.16, 9.45)	0.7365	0.7438
								
	A3 vs A1	1	-	Inf (NaN, Inf)	0.7327	Inf (NaN, Inf)	0.4292	0.1666
								
Disease Location	L2 vs L1	1	-	NaN (NaN, NaN)	0	NaN (NaN, NaN)	0	0.4389
								
	L3 vs L1	1	-	0 (0, NaN)	0.0300	0 (0, NaN)	0.0757	0.0906
								
Disease Behavior	B2 vs B1	1	-	Inf (NaN, Inf)	0.6396	Inf (NaN, Inf)	0.7511	0.5913
								
	B3 vs B1	1	-	0.03 (0, 0.34)	0.0020	0.04 (0, 0.47)	0.0155	0.0078
								
Surgery	Yes vs No	1	-	0.1 (0.02, 0.63)	0.0236	0.3 (0.05, 1.88)	0.3706	0.5764
								

Inf: infinity.

NaN: Not a number.

### rs207959 Genotype Affects SLCO3A1 Expression in CD Patients and CD Patients have Increased SLCO3A1 Expression Compared with Controls

**Figure 1:**
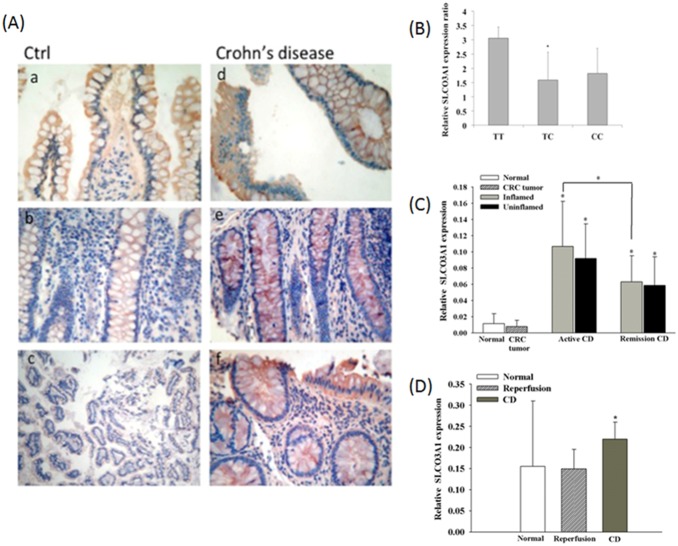
rs207959 T/C elevates SLCO3A1 mRNA and protein expression levels in colon and small intestine tissue of normal, non-CD diseases, and CD patients. (A) Expression of SLCO3A1 in colon tissue of normal (non-tumor portion of colorectal cancer patients) and CD patient by immunohistochemical staining (400X). (B) Expression of SLCO3A1 in colon tissue of different CD patient genotypes (n = 3 for TT/CC group and n = 10 for TC group) determined by quantitative PCR. (C) Expression of SLCO3A1 mRNA in colonic tissue (n = 30 for normal and colorectal cancer tumor; n = 24 for active CD patients; n = 6 for remission CD patients) determined by quantitative PCR. (D) Expression of SLCO3A1 mRNA in small intestine tissue (n = 3 for normal; n = 5 for reperfusion inflammation; n = 6 for active CD patients) determined by quantitative PCR (*P<0.05).

Increased SLCO3A1 expression was observed in intestinal epithelial cells of CD patients compared to normal controls based on IHC staining (Figure 1A). To further evaluate the relationship between the rs207959 (T) allele and CD, we investigated SLCO3A1 mRNA expression in colonic tissue of CD patients. As shown in Figure 1B, patients with genotype TT had approximately 2-fold higher SLCO3A1 mRNA expression than genotypes TC and CC, as determined by quantitative PCR. Next, we compared SLCO3A1 mRNA expression in normal and non-CD diseases (colorectal cancer for colonic expression and reperfusion inflammation for small intestine expression) with CD patients (Figure 1C and 1D). We found that in both colon and small intestine, CD patients had significantly increased mRNA expression of SLCO3A1 compared with the non-CD diseases and normal controls. Furthermore, active CD had significantly increased expression of SLCO3A1 compared with remission CD (Figure 1C), suggesting that SLCO3A1 plays a role in CD.

### Overexpression of SLCO3A1 Increases NF-κB Activation and Enhances Phosphorylation of ERK and JNK but not P38 and AKT

**Figure 2:**
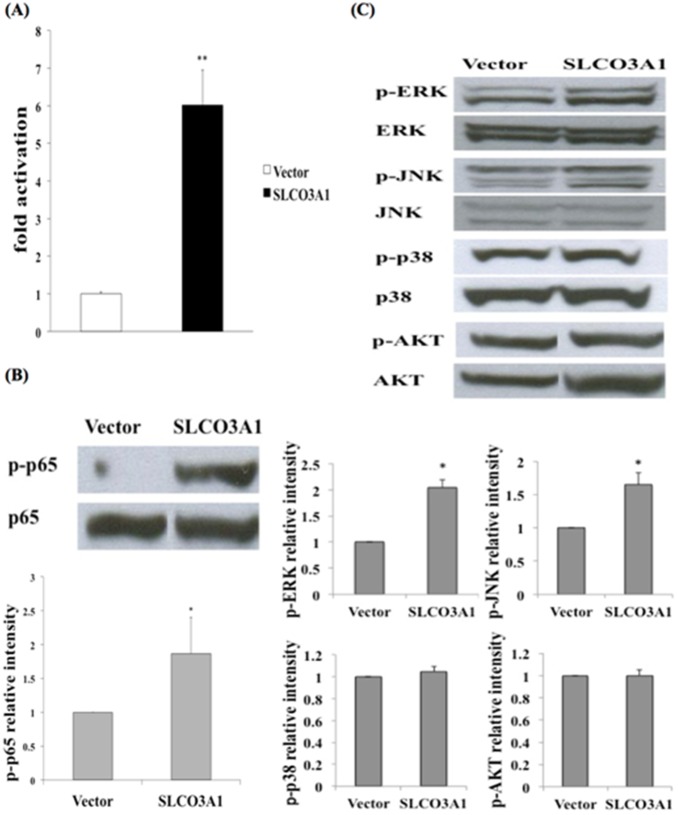
Overexpression of SLCO3A1 induces NF-κB activation, enhances the phosphorylation of two classes of MAPKs (ERK and JNK), and augments NF-κB activity. (A) Overexpression of SLCO3A1 induced approximately 6-fold higher NF-κB activation (**P<0.01). (B) Overexpression of SLCO3A1 in HEK293T cells resulted in increased p65 expression (*P<0.05). (C) ERK and JNK expression increased with overexpression of SLCO3A1, while expression of p38 and AKT showed no difference (*P<0.05). All experiments were performed at least 3 times.

Since increased expression of SLCO3A1 was observed in CD patients compared with the control group, we examined the role of SLCO3A1 in the inflammatory process by overexpressing SLCO3A1 in HEK293T cells. Overexpressing SLCO3A1 led to an increase in NF-κB activation, approximately 6-fold higher compared to the vector control, as seen by a NF-κB reporter luciferase assay (Figure 2A). Western blot analysis also revealed increased phosphorylation of the NF-κB p65 subunit in cells overexpressing SLCO3A1 (Figure 2B). In addition to the NF-κB pathway, we assessed phosphorylation of components of the MAPK and AKT pathways, which also play a role in inflammation. Overexpression of SLCO3A1 increased phosphorylation of ERK and JNK, while phosphorylation of p38 and AKT were unchanged (Figure 2C).

### Nicotine Augments the Activation of NF-κB Activity in Cells Overexpressing SLCO3A1

**Figure 3:**
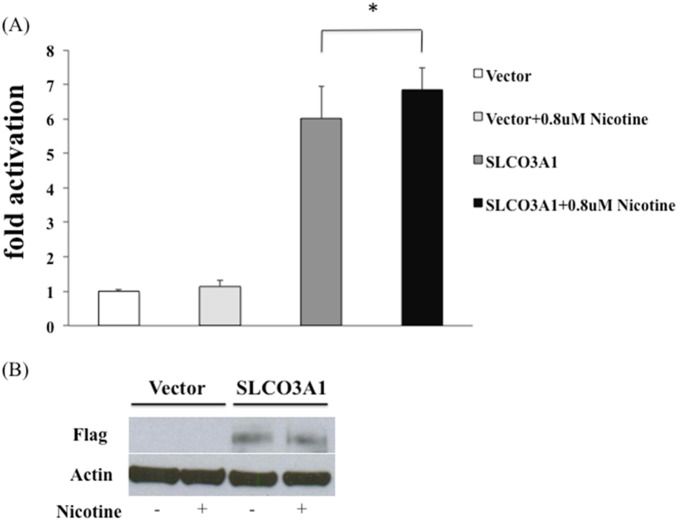
Activation of NF-κB by nicotine in SLCO3A1 overexpressing cells. (A) HEK293T cells were transfected with the NF-κB reporter plasmids, together with an empty vector or SLCO3A1 constructs. Addition of 0.8 µM nicotine for 24 hours resulted in increased NF-κB activity in cells overexpressing SLCO3A1 (*P<0.05). (B) Western blot analysis from cell lysates demonstrates equal transfection efficiencies.

Since smoking is known to be an aggravating factor for CD and SLCO3A1 was reported to be associated with nicotine dependence [18]–[21], we evaluated the effect of nicotine on NF-κB activation in cells overexpressing SLCO3A1. Addition of nicotine (0.8 µM) to cells overexpressing SLCO3A1 resulted in a further significant increase in NF-κB activation compared to addition of DMSO as control (Figure 3). This finding suggests that nicotine may augment the NF-κB activity/inflammatory process in CD patients.

## Discussion

Using a two-stage approach of Illumina platform-based and Sequenom MassArray analyses, along with functional studies, we identified a new CD-associated gene in Taiwanese patients. The SNP rs207959, located in an intron of *SLCO3A1* (also known as OATPD, OATP-D, OATP3A1, FLJ40478, and SLC21A11) at 15q26, showed a significant association with CD in the two-stage analysis. Our study showed that rs207959 allelic differences correlate with altered SLCO3A1 mRNA expression. Patients with genotype TT exhibit greater expression than patients with genotype TC or CC, demonstrating that the rs207959 T/C change affects the mRNA expression of SLCO3A1. In other systems it has been shown that transcriptional regulatory elements in introns and the intronic elements may determine alternative splicing patterns and thereby regulate biological functions [22]–[24].

Solute carrier organic anion transporters (SLCOs/OATPs) are multispecific transport proteins that are widely expressed in many tissues in the body. They mediate the Na^+^-independent uptake of large amphipathic organic anions [25]. The solute carrier organic anion transporter family member 3A1 (SLCO3A1) is one of the uptake transporters that belongs to the solute carrier family [26]. SLCO3A1 protein was recently detected in the epithelial tissues of lactiferous ducts in normal breast tissue [27]. We have shown that SLCO3A1 is also expressed in the intestinal epithelium, where both mRNA and protein expression are significantly increased in CD patients compared with normals and individuals with non-CD disease. However, it is still unclear how SLCO3A1 influences cell functions.

By comparing the allelic frequency and calculating the OR of three different genotypes in CD patients, we also found that patients with genotype TT were more susceptible to CD compared to patients with genotype TC or CC. More specifically, patients with genotype TT have a greater risk of gastrointestinal tract perforation than patients with genotype TC or CC. From our results, we also observed that active lesions showed increased expression of SLCO3A1 compared to scar tissue, supporting the hypothesis that SLCO3A1 expression correlates with disease activity and outcomes for CD patients.

NF-κB is a key pro-inflammatory transcription factor and controls many genes involved in the inflammatory process [28]; activation of NF-κB is pivotal in pro-inflammatory signal transduction [29]. Previous studies have shown that NF-κB regulates the IBD inflammatory process and is activated in mononuclear cells of the intestinal lamina propria in CD patients [29]. In our study, we found that SLCO3A1 mediates inflammation by activating NF-κB transcriptional activity. Moreover, SLCO3A1 induces stronger activation of ERK and JNK phosphorylation, leading to a more intense and protracted NF-κB activation.

Smoking has long been considered a risk factor for CD. CD patients who smoke suffer more clinical relapses and undergo more operations than nonsmoking CD patients [18], [19]. One study has shown a correlation between the SNP of SLCO3A1 and QT prolongation in schizophrenic patients treated with iloperidone [30]. Schizophrenia is known to be associated with a high prevalence of smoking [20]. Meta-analyses of GWAS have also shown that SLCO3A1 is associated with nicotine dependence [21]. As shown in a previous study, nicotine increases oxidative stress, activates NF-κB, and induces apoptosis [31]. In our study, we have provided evidence that nicotine induction leads to enhanced NF-κB activation in the presence of SLCO3A1. These findings are consistent with the hypothesis that smoking exacerbates the course of CD due to allelic change of *SLCO3A1*.

We are aware of limitations associated with this study due to the small sample size. As emphasized in the introduction, CD is still a low incidence/prevalence disease in Taiwan. Therefore, despite our best efforts to enroll patients, the sample size was small. We would like to see further validation of these results from other countries in the future. Secondly, the tissues used for control were not ideal. Theoretically, age-and sex-matched healthy individuals and subjects with non-IBD inflammation (e.g., diverticulitis or gastroenteritis) would be more appropriate for the control group. However, in clinical practice, these conditions are not appropriate or indicated for endoscopy or surgery. Therefore, we were not able to obtain this tissue for use as control. Instead, we used colorectal cancer and intestine transplantation tissue since these are obtainable in clinical practice. The gender ratio of colorectal cancer is similar to that of IBD in Taiwan [32]. With respect to age, we performed a correlation analysis of the expression of SLCO3A1 and age, and found no correlation between them (data not shown). Therefore, we concluded that it would be acceptable to use the current control for interpreting the results.

In conclusion, *SLCO3A1* is a novel CD-associated gene based on our Illumina platform analysis and functional study results. Expression of SLCO3A1 activates the NF-κB transcription factor mediating inflammatory processes, consequently inducing increased activation of ERK and JNK phosphorylation and leading to a more intense and protracted NF-κB activation in intestinal epithelial cells. Active disease CD tissue expressed higher levels of SLCO3A1 compared with tissue analyzed from patients in remission. Stronger inflammation is associated with a greater chance of a perforated CD phenotype. Nicotine enhances the NF-κB activation in the presence of SLCO3A1, which can partially explain smoking’s influence as an aggravating factor for CD.
